# Supplementary material for: Positioning of negative feedback loops within immune signaling pathways influences gene expression noise
Source: bioRxiv. 2025 Jun 6:2024.02.22.581613. Preprint. [Version 2] doi: 10.1101/2024.02.22.581613 (PMC12157479; doi:10.1101/2024.02.22.581613)
Supplement: Supplement 2 [file NIHPP2024.02.22.581613v2-supplement-2.pdf]

bioRxiv preprint doi: https://doi.org/10.1101/2024.02.22.581613; this version posted June 6, 2025. The copyright holder for this preprint (which was not certified by peer review) is the author/funder, who has granted bioRxiv a license to display the preprint in perpetuity. It is made available under aCC-BY 4.0 International license.

Supporting information

S1 Text. Supplementary information file, including a detailed description of the Gillespie algorithm and the deterministic model. The first section contains a detailed description of simulations of Imd and Toll signaling using the Gillespie algorithm. The second section shows the system of ODE modeling deterministic signaling pathways.

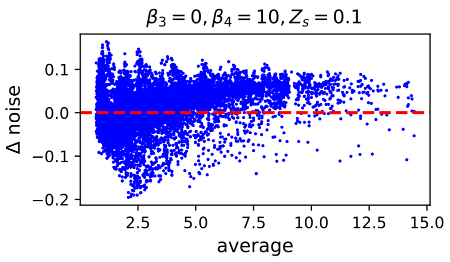

Fig.S1. In Imd signaling, upon strong binding of the downstream NFL to the promoter of AMP genes, AMP noise is suppressed only when gene expression is small. The change in noise is shown for a downstream biased regime with a strong expression of the downstream NFL ($\beta_4 = 10$) and 10x stronger binding ($Z_s = 0.1$) compared to Fig.2 is compared to a circuit with no NFL. The comparison is done for parameters with the same average AMP expression.

bioRxiv preprint doi: https://doi.org/10.1101/2024.02.22.581613; this version posted June 6, 2025. The copyright holder for this preprint (which was not certified by peer review) is the author/funder, who has granted bioRxiv a license to display the preprint in perpetuity. It is made available under aCC-BY 4.0 International license.

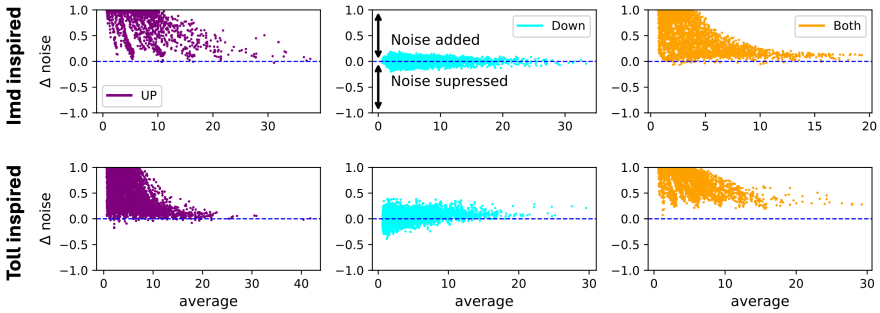

Fig.S2. For a high bacterial proliferation rate, the downstream NFL reduces AMP noise for some parameters, while the upstream NFL amplifies noise. The change in noise across three regimes (upstream biased, downstream biased, both from left to right) compared to a circuit with no NFLs. The comparison is done for parameters with the same average AMP expression. Here, the bacterial proliferation is set to k_0 =0.5.

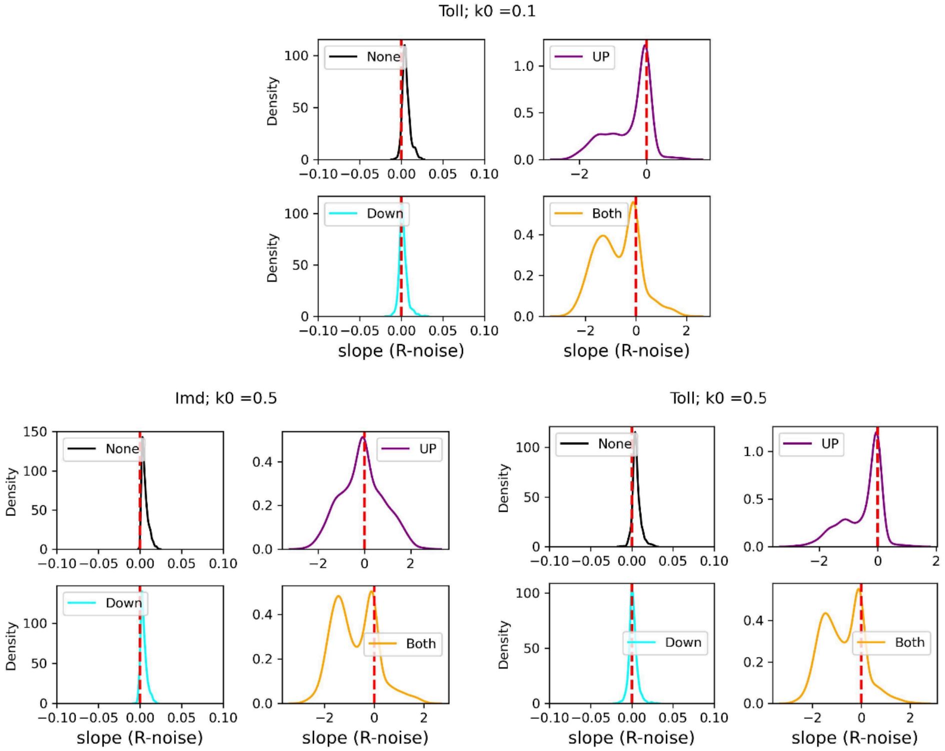

Fig.S3. The combination of the upstream NFL and a strong expression of PFL reduces AMP noise. The slope between receptor activation rate and AMP noise is plotted for Toll signaling ($k_0 = 0.1$) at the top, Imd signaling ($k_0 = 0.5$) at the bottom left, and Toll ($k_0 = 0.5$) at the bottom right. Positive slope indicates that increasing receptor activation rate increases noise, whereas negative values indicate that increasing the receptor activation rate reduces noise.

bioRxiv preprint doi: https://doi.org/10.1101/2024.02.22.581613; this version posted June 6, 2025. The copyright holder for this preprint (which was not certified by peer review) is the author/funder, who has granted bioRxiv a license to display the preprint in perpetuity. It is made available under aCC-BY 4.0 International license.

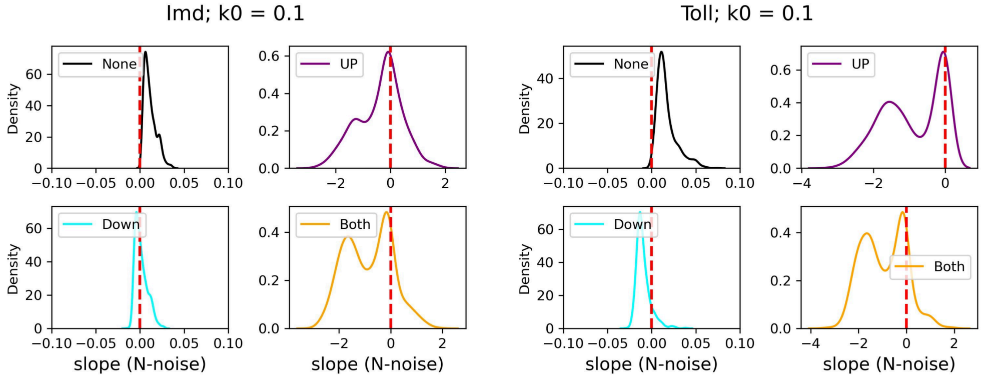

Fig.S4. The combination of the upstream NFL and a strong expression of the positive regulator (NF-κB) reduces AMP noise. The slope between NF-kB activation rate and noise is plotted for Imd signaling on the left and Toll signaling on the right. Positive slope indicates that increasing NFkB activation rate increases noise, whereas negative values indicate that increasing NFkB activation rate reduces noise. Here, we set $k_0 = 0.1$.

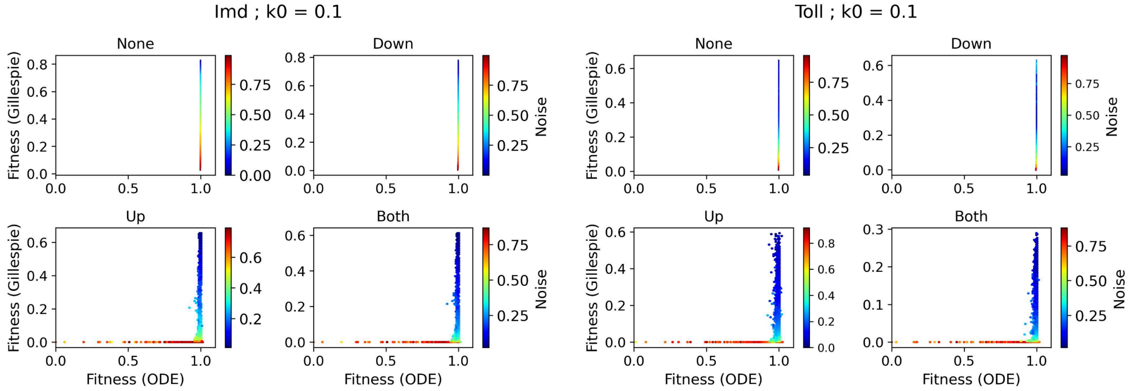

Fig.S5. Noise reduces fitness even for optimal parameters (high fitness in the ODE model). Fitness (e^{-B}) values for different combinations of background parameters are shown using the Gillespie algorithm (Y-axis) and the ODE system (X-axis). The heatmap color represents the level of noise. Four parameter regimes are shown: No NFL, downstream NFL only (Down), upstream NFL only (Up), and both NFLs. To enhance the visibility of the pattern, the noise values are log-normalized. Imd signaling results are shown on the left, and the results for Toll signaling are on the right. For each pathway, in the top two panels, the fitness of the ODE system is always near 1, regardless of the background parameter value. Here we set $k_0 =$ 0.1.

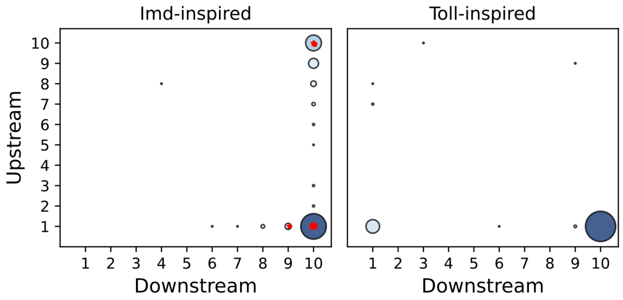

bioRxiv preprint doi: https://doi.org/10.1101/2024.02.22.581613; this version posted June 6, 2025. The copyright holder for this preprint (which was not certified by peer review) is the author/funder, who has granted bioRxiv a license to display the preprint in perpetuity. It is made available under aCC-BY 4.0 International license.

Fig.S6. When bacterial proliferation is high, immune signaling pathways evolve either towards a downstream NFL or both NFLs. Evolution of Imd (left) and Toll signaling when bacterial proliferation rate is high ($k_0 = 0.5$). Plots show the final values of parameters controlling downstream (X-axis) and upstream (Y-axis) NFLs after 5,000 generations. Stars show simulations in which noise increases across generations. The size of the circles and the darkness of the color for every combination of NFLs are determined by the proportion of simulations that converge to that combination of NFLs.

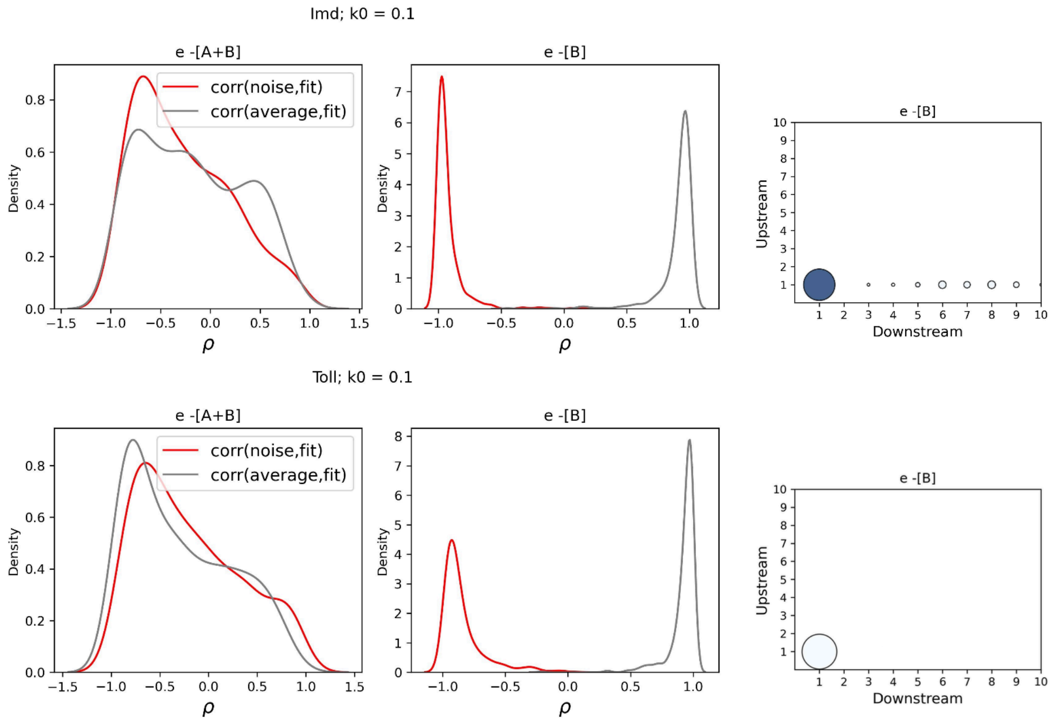

Fig.S7. Noise only evolves when signaling pathways must reduce the cost of AMP expression. The correlation plots on the left are shown for e-[A+B] and e-[B] fitness functions. Here, we measured the Spearman correlation between noise (and average expression) and fitness across 5,000 generations of Imd and Toll evolution. Since fitness only increases during the evolution of both pathways (i.e., mutations that decrease fitness are rejected), a positive correlation with fitness indicates that the parameters (noise or average expression) is increasing across generations, whereas a negative correlation means the parameter is decreasing as the evolution continues. Here, we only see negative correlation for noise values (red plots) when we have e-[B]. On the other hand, average expression shows positive correlation when we have e-[B] to eliminate the pathogen. The results for e-[A+B] is more complicated because the signaling pathway should minimize both B and A. Minimizing A increases noise, and results in positive correlation values in some simulations when we have e-[A+B]. On the right the final combinations of upstream (Y-axis) and downstream (X-axis) NFLs are shown after 5000 generations when we have e-[B]. For all simulations presented here, we set $k_0 = 0.1$.

bioRxiv preprint doi: https://doi.org/10.1101/2024.02.22.581613; this version posted June 6, 2025. The copyright holder for this preprint (which was not certified by peer review) is the author/funder, who has granted bioRxiv a license to display the preprint in perpetuity. It is made available under aCC-BY 4.0 International license.

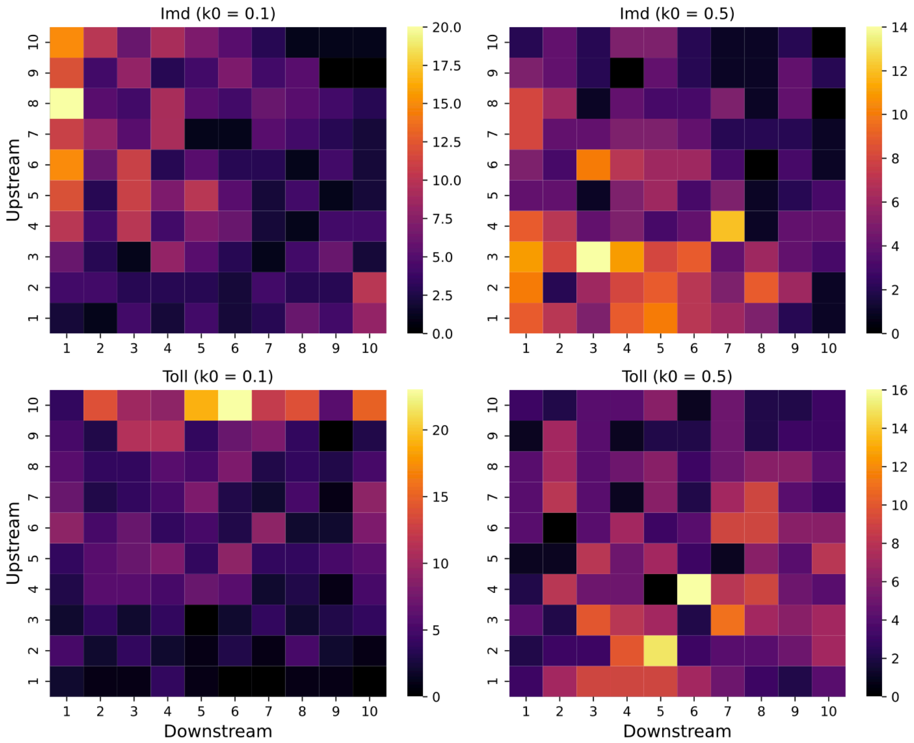

Fig.S8. For a lower bacterial proliferation rate, both Imd and Toll signaling evolve toward an upstream-biased NFL. The evolution of a deterministic Imd (on the top) and Toll (on the bottom) signaling for two bacterial proliferation rates (k_0=0.1 on the left and k_0=0.5 on the right). The brighter colors show combinations of downstream and upstream NFLs that evolve more often.
